# *HBP Builder*: A Tool to Generate Hyperbranched Polymers and Hyperbranched Multi-Arm Copolymers for Coarse-grained and Fully Atomistic Molecular Simulations

**DOI:** 10.1038/srep26264

**Published:** 2016-05-18

**Authors:** Chunyang Yu, Li Ma, Shanlong Li, Haina Tan, Yongfeng Zhou, Deyue Yan

**Affiliations:** 1 School of Chemistry and Chemical Engineering, State Key Laboratory of Metal Matrix Composites, Shanghai Jiao Tong University, 800 Dongchuan Road, Shanghai 200240, P. R. China

## Abstract

Computer simulation has been becoming a versatile tool that can investigate detailed information from the microscopic scale to the mesoscopic scale. However, the crucial first step of molecular simulation is model building, particularly for hyperbranched polymers (HBPs) and hyperbranched multi-arm copolymers (HBMCs) with complex and various topological structures. Unlike well-defined polymers, not only the molar weight of HBPs/HBMCs with polydispersity, but the HBPs/HBMCs with the same degree of polymerization (*DP*) and degree of branching (*DB*) also have many possible topological structures, thus making difficulties for user to build model in molecular simulation. In order to build a bridge between model building and molecular simulation of HBPs and HBMCs, we developed *HBP Builder*, a C language open source HBPs/HBMCs building toolkit. *HBP Builder* implements an automated protocol to build various coarse-grained and fully atomistic structures of HBPs/HBMCs according to user’s specific requirements. Meanwhile, coarse-grained and fully atomistic output structures can be directly employed in popular simulation packages, including *HOOMD*, *Tinker* and *Gromacs*. Moreover, *HBP Builder* has an easy-to-use graphical user interface and the modular architecture, making it easy to extend and reuse it as a part of other program.

Hyperbranched polymers (HBPs) as a polymer with a three-dimensional and highly branched globular structure have emerged during the past twenty years^1^,^2^,^3^,^4^. Unlike well-defined dendrimers, HBPs have linear units in structures except dendritic and terminal units, and the degree of branching (*DB*) of them are usually less than 0.5. So, HBPs are not as regular as dendrimers. However, HBPs also have the similar properties to dendrimers when compared with the linear counterparts, including a large population of functional groups, lower solution or melt viscosity, and better solubility^4^,^5^. In addition, the synthesis of HBPs generally involves one-pot reaction without the troublesome and time-consuming separation and purification process like dendrimers^6^,^7^,^8^,^9^,^10^,^11^, which has greatly reduced the cost. Thus, HBPs have attracted great interest from both the scientific and industrial communities, and have found many applications in various fields such as coatings^12^,^13^, additives^14^,^15^, drug delivery^16^, supramolecular chemistry^17^,^18^, and so on. In recent year, amphiphilic hyperbranched multi-arm copolymers (HBMCs) (see Figure S1 in supplementary information) consisting of a hyperbranched core and many linear arms have been proved to be excellent self-assembly precursors, and many delicate supramolecular structures with all scales and dimensions have been prepared through the solution self-assembly of amphiphilic HBMCs^16^,^19^,^20^,^21^,^22^.

Although the rapid progress has been made in experimental studies of HBPs and HBMCs, there are still many problems such as the static and dynamic properties of HBPs/HBMCs at micro level have not been fully understood due to the limitation of experimental means. For example, the conformational behavior of individual HBPs/HBMCs in solution, the interaction mechanism between HBPs/HBMCs and small molecules or polymers (especially the biopolymers like DNAs or RNAs), and the conformation and phase separation behaviors of individual HBMCs during the self-assembly processes *et al.*^16^,^21^,^22^. Thus, it is necessary to resort to the theoretic calculations or simulations to fully study HBPs and HBMCs.

In fact, nowadays, computer simulation has been becoming a powerful tool to investigate molecular information at any required level and has been a necessary complementary to experimental method. Different simulation methods were developed for different length and time scales. Ab initio molecular dynamics were developed to study complex processes at the electronic level, including the breaking and making of chemical bonds, by means of purely electronic structure theory^23^,^24^. The classical all-atom (AA) simulation methods^25^,^26^,^27^,^28^,^29^,^30^, which describe the atomic-level detail, were used to simulate the conformation and dynamical behaviors of polymers. Due to the current computational power, AA simulations are often constrained in time scale up to 100 ns and limited in the number of atoms. Thus the coarse-grained (CG) methods have been developed to simulate the aggregate behaviors of large number of molecules in bulk or in solution^31^,^32^,^33^,^34^. However, polymers are complex macromolecules whose spatial scales can vary from the atomistic level to the scale of tens of nanometers. The corresponding time scales of the dynamic processes relevant for different materials properties span an even wider range, from femtoseconds to milliseconds, seconds or for large scale ordering processes such as phase separation in blends. The spatial and temporal scales become even larger when the polymers form aggregates during the self-assembly process. Thus, no single model or simulation algorithm currently available can encompass this range of length and time scales. To address this issue, some multiscale molecular modeling methods have been developed and applied to simulate polymers^35^,^36^,^37^,^38^,^39^.

Although significant progresses have been made for simulation methods, considering the special molecular structure of HBPs/HBMCs, the simulation studies of them are still quite limited. In order to perform the computer simulations on HBPs/HBMCs, the first necessary step is model building, which is quite difficult. A famous dendrimer builder toolkit (DBT) for generating dendrimer configuration of desired generation and with various architectures was developed by Vishal Maingi and co-workers^40^. However, unlike these monodisperse polymers, HBPs are a mixture of thousands of different topological polymers. In experiments, for both HBP solution and bulk, it is a mixture of many HBP molecules with a polydispersity of molecular weight, a polydispersity of DB, and a distribution of topological structure. Furthermore, it is even more difficult to describe HBMCs, which should not only contain the three structure parameters as mentioned above for conventional HBPs, but also should include the distributions of the length of the arms (polydispersity of molecular weight), the number of the arms (graft ratio), and the graft position. It is very challengeable to set up a general toolbox to fully describe HBPs and HBMCs.

So far, due to the very complex topologic structures, most of the reported theoretic models on HBPs/HBMCs are based on coarse-grained method. In summary, up to now, there are three types of theoretic models. The simplest type of them is to regard HBPs/HBMCs as the monodisperse polymers. For example, in the dissipative particle dynamics (DPD) simulations on the self-assembly of amphiphilic HBMCs, all the polymers have the same predetermined hyperbranched structure in the simulation box^41^,^42^. This monodisperse model is quite simple but is far from the fact. The second type of the models involves building HBPs randomly and then selecting several representative polymers for further study. The sequential addition method is the representative method of this type. This method was first developed for building HBPs to simulate its intrinsic viscosity with varying topologies by Davies *et al.*^43^. Since then, this method was widely used to investigate the conformational behavior of pure HBPs and HBP complexes. For example, HBPs under shear flow^44^,^45^ and elongational flow^46^, the conformational behavior of charged/uncharged HBPs in solution^47^ and the influence of the Wiener index^48^ on the intrinsic viscosity and radius of gyration^49^ were investigated by Adolf and Karatasos *et al.* Nevertheless, these models are still deviated from the actual polydisperse HBPs. The third type of models has sincerely considered the polydispersity of HBPs, and was developed by Ricardo Rodríguez Schmidt and coworkers^50^,^51^. In this model, they firstly obtained the coarse-grained model parameters from atomic-level simulations of small chains fragments, and then they used the Monte Carlo technique to generate a set of polydisperse HBPs with required *DBs*. This multi-scale simulation method has the advantage of investigating conformational and dynamic behavior of HBPs by a very simple coarse-grained model and none of the parameters of that model need to adjust to fit experimental data.

Although the abovementioned three coarse graining methods are efficient and can allow the exploration of the behavior of HBPs/HBMCs over longer times, the loss of the atomistic information is inevitable. However, in many cases atomistic insight is required when special interactions need to be considered, for example, the noncovalent interactions like hydrogen bonds or specific chemical interactions among polymers when the HBPs or HBMCs are in the self-assembly process^17^,^18^. In addition, in the biomedical applications of HBPs/HBMCs or their self-assemblies, the interactions between them and drugs, DNAs or proteins should have an atomistic resolution^16^. Moreover, electrostatic interaction play a very important role in the budding and fission process of HBPs/HBMCs vesicle^52^,^53^, the atomistic resolution are essential for recognizing the microscopic regulating mechanism. Therefore, it is very useful to combine coarse-grained and atomistic methods together in the computer simulations on HBPs/HBMCs.

Based on the above experimental and theoretical studies, there are two crucial problems need to be considered for developing a precise model for HBPs and HBMCs. On the one hand, the polydispersity of HBPs and HBMCs should be fully taken into account, including the polydispersity of molecular weight, DB, and distribution of topological structure; on the other hand, developing computational model for HBPs and HBMCs should combine coarse-grained and all-atom models together, especially in the case where both the aggregated and unimolecular information are important. However, to our knowledge, up to now no theoretic toolbox with all these functions has been reported for HBPs/HBMCs.

To address this issue, we developed *HBP Builder*, a C language open source HBP/HBMC building toolkit. *HBP Builder* implements an automated protocol to build coarse-grained and fully atomistic structures of HBPs/HBMCs according to user’s specific requirements. The *HBP Builder* program mainly includes two function modules: one of them is *HBP Builder_CG*, which is developed for quickly generating thousands of polydisperse coarse-grained HBPs and HBMCs with required average PDI, DB and degree of polymerization (DP); the other is *HBP Builder_AA*, which is developed for converting coarse-grained HBPs and HBMCs to fully atomistic structures. In order to improve operation, we designed an easy-to-use interactive graphic interface for each function module and thereby the characteristic parameters (including PDI of hyperbranched core and linear arm unit, DP of core and arm, and DB *et al.*) can be easily defined via keyboard input. Moreover, various types of output files can be generated from *HBP Builder* for the direct use by DPD/CGMD and all-atom simulations, for example used by HOOMD, Tinker and Gromacs simulation packages. Due to the modular architecture of *HBP Builder*, it can be easier extended and reused it as a part of other programs. The design ideas and implementation process of each function module in *HBP Builder* will be described explicitly by building various topological structures of HBPs/HBMCs molecules. The remainder of this article is organized as follows. Section 2 respectively presents the design ideas and application examples of *HBP Builder_CG* for generating coarse-grained HBPs and HBMCs. In section 3, the basic principle and application examples for converting coarse-grained HBPs/HBMCs to fully atomistic structures in *HBP Builder_AA* module are given. The performances of *HBP Builder* are given in section 4. Finally, conclusion and outlook for *HBP Builder* are stated in section 5.

## The ***HBP Builder_CG*** function module

The *HBP Builder_CG* module mainly realized two functions: one is to generate coarse-grained HBPs according to user required PDI, DP and DB; the other is to build HBMCs based on the generated HBPs. The generation process of HBPs and HBMCs are respectively described in more detail below.

### The generation of HBPs

Unlike monodisperse polymers, HBPs are a mixture of thousands of different topological polymers. In the actual process of experiments not only the molecular weight has polydispersity, but even with same DB and DP also has many possible topological structures. So investigating the properties of HBPs through monodisperse model is obviously not appropriate. Thus, constructing polydisperse HBPs to represent the real system according to the parameters measured from experiment is one of main function of *HBP Builder_CG* module.

**Figure 1:**
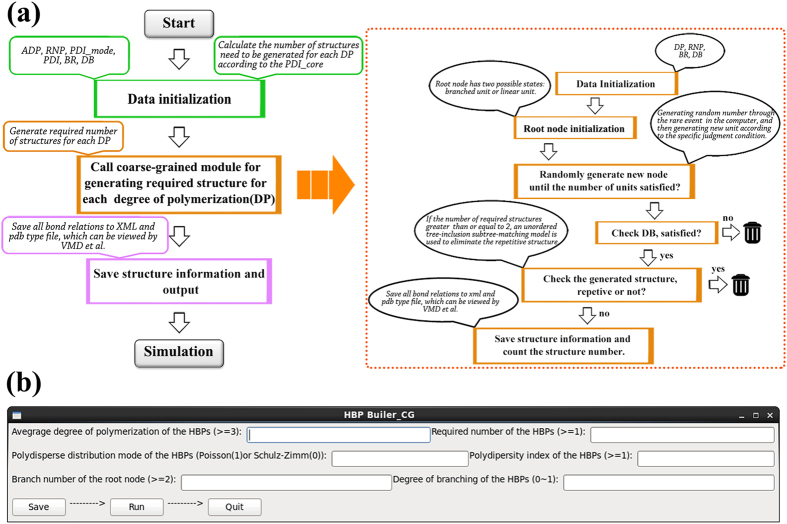
The flow chart of *HBP Builder_CG* module for generating HBPs. (**a**) The interactive input graphic interface of *HBP Builder_CG* module (**b**).

In Fig. 1a, we present the flowchart to show the generation process of coarse-grained HBPs through the *HBP Builder_CG* module. This flowchart was made of three subprograms: the input setting of the characteristic parameters, the coarse-grained HBPs generation, and the output of HBPs structures. The detailed descriptions for each subprogram are in the following sections.

### Input setting of the characteristic parameters

The interactive graphic interface of *HBP Builder_CG* defines the characteristic parameters of hyperbranched system, as shown in Fig. 1b. User can control over the topological structures of the output file through it. The specific parameter setting and description are as follows.

*Average degree of polymerization of the HBPs* (ADP) is the average DP of the required HBPs, which should be at least greater than or equal to 3.

*Required number of the HBPs* (*RNP*) is the number of HBPs needs to be generated.

*Polydisperse distribution mode* (PDI_mode) and the *polydispersity index* (PDI) of the HBPs describe the distribution mode and the distribution width of molecular weight. In order to represent the wide molecular weight distribution in cationic polymerization processes, Schulz-Zimm and Poisson distributions were utilized in *HBP Builder_CG*, respectively, to construct our polydisperse hyperbranched systems. The Schulz-Zimm distribution obeys the form of 54,55,56:


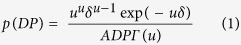


where δ = *DP*/*ADP*. The *PDI* for this distribution is *PDI* = (*u*+1)/*u*. The Poisson distribution is specified by^54^:


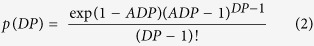


the *PDI* for Poisson distribution is *PDI* = 1 + (*ADP* − 1)/*ADP*^2^. As a menu setting in *HBP Builder_CG*, user can freely choose Schulz-Zimm or Poisson distributions for HBPs.

*Branch number of the root node* (BR) is the graft number of the root node, which should be greater than 1.

*Degree of branching of the HBPs* (DB) describes the statistical DB of HBPs.

Moreover, generally speaking, DP and DB are two characteristic parameters to describe a specific hyperbranched structure, which is defined as^57^:


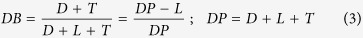


In Equation (3), D, L and T are the number of dendritic unit, the number of linear unit and the number of terminal unit respectively. In fact, only DB and DP cannot fully describe the connectivity of HBPs, because it is obviously that one HBP has many connection ways for same DB and DP. Therefore, we also employed Wiener index (WI)^56^ to characterize different hyperbranched structures in our program. WI is defined as the cumulative distance between all pairs of units within the molecules measured in terms of the number of intervening bonds between two units. Herein it is calculated according to the following expression^58^,^59^.


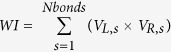


In Equation (4), *V*_*L,s*_ and *V*_*R,s*_ are the number of units in the left and that in the right of the center of each bond s. As reported by Karatasos *et al.*^59^, for each DB and DP, low WI describes a relatively compact dendrimer-like structure and one with a relatively high WI describes a less compact, closer to a star-like shape. In other words, structures with lower WI seem to develop their branches closer to their inner core, while structures with higher WI are branched toward the periphery. Once *HBP Builder_CG* run has finished, it will list all the structures’ WI in the output file, user can select the interested structures for further investigation.

### The generation process of Coarse-grained HBPs and output

As shown in Fig. 1a, in the practical generation process, we firstly calculated the number of HBPs needs to be generated for each *DP* according to the ADP, PDI and RNP. Then the subprogram of coarse-grained structure generation will be called to build HBPs for each DP. In this section, we will introduce the concrete realization of coarse-grained HBP generation.

**Figure 2:**
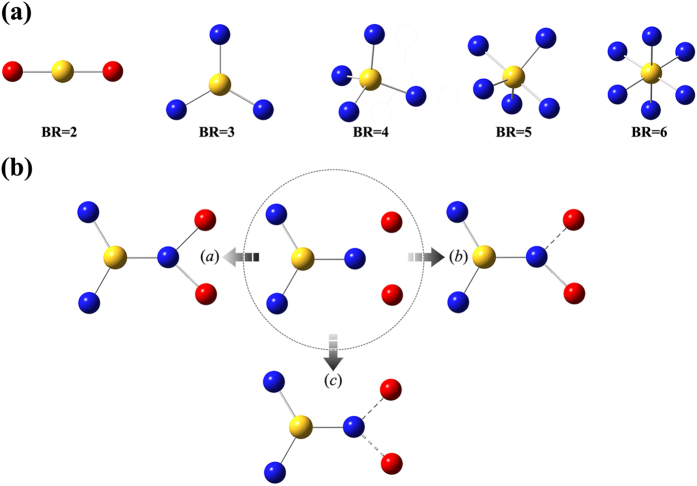
The initialization of the root node for linear and branched structures. (**a**) The diagram of the addition rules (**b**). The full line represents successfully added; the dotted line represents unsuccessfully added. Yellow bead represents root unit; blue bead represents the initialized units; red bead represents the new added units.

Based upon the graft number of the root node defined in the input window, *HBP Builder_CG* initializes root unit firstly. For example, if assign 2 to the branchpoint of root node, it will generate a linear structure. Otherwise it will generate a branched structure. As shown in Fig. 2a, the root unit has several possible states. However, initialize which one depended on the *BR* value of user’s input. After the initialization of root node is complete, we built HBPs with desired DP from the initial unit. The added process of the next DP-1-BR units will accord to the specific random judgment condition. The random number will be generated through the random event in the computer. The addition process of new units is from inner to the exterior and the new units are added one after another until the required DP is reached. In order to clearly explain the added rule utilized in *HBP Builder_CG* module, we take Fig. 2b as an example to illustrate. Firstly, two chances to generate and judge random number were given to each to be connected unit. Then there are three possible cases appear after that: (a) successfully added two new units; (b) successfully added one new unit; (c) no new unit added. For the first two cases, *HBP Builder_CG* will go to the next unit and repeat this addition process. But for the last case, *HBP Builder_CG* will give this unit another two added chances until at least one new unit successfully added in this position. It should be noted that we have done a good deal of work about the method to judge new unit addition. After a lot of attempts, we classify the judgment conditions into two cases: DB greater than 0.5 and DB less than or equal to 0.5. And different approaches are used in different cases. The concrete realization in the program is as follows:

*if* (*db*<=*0.5 && db* >=*0*) *randb *= (*int*)/(*1.0/db*);

*else if* (*db*>*0.5 && db*<=*1.0*) *randb* = (*int*)(*1.0*/(*1.0-db*));

*if* ((*db*>*0.5 && rand*()*%randb!*=*0*||(*db*<=*0.5 && rand*()*%randb* = *0*) *added a new node…*

A large number of trails have shown that the above judgment method for generating required structure is effectual and time-saving. Next, we compute DB of the generated structure when DP reaches the set value. If the deviation between the real DB and that of the set value is in an acceptable range (user can set the tolerance value in the program), *HBP Builder_CG* will save the coarse-grained structure. Otherwise, if the deviation exceeds the range of accuracy or the number of required HBPs is not reached, *HBP Builder_CG* will repeat the above generation process.

It is important to note that a method to judge repetitive structure is required when the number of HBPs is greater than 1. Herein, an unordered tree-inclusion subtree-matching model was employed in *HBP Builder_CG* program to remove the repetitive structure. In the matching model, each HBP could be seen as an unordered tree grows from the root node. And each unit of HBPs can be viewed as a node of the tree. Then each subtree of two unordered tree will be compared by using a recursive method. If there is a one-to-one correspondence between each subtree, it means that these two structures are equivalent. Then the information of the repetitive structure will be discarded and the above generation process will be executed repeatedly. Through the repeated verification, the unordered tree-inclusion matching model has proved to be a precise and efficient way for discarding the repetitive structures.

After all the required HBPs has been successfully generated, *HBP Builder_CG* will save molecular structure information for each HBP and packed all the molecules into one simulation box. Furthermore, the solvent molecules can be added by calling *PACKMOL* software^60^. Finally, we respectively saved the output file to xml type for DPD simulation (employed by *HOOMD* program package) and gro (and itp) type for coarse-grained simulation (employed by *Gromacs* program package).

### The application examples for Coarse-grained HBPs

To illustrate the practicality of *HBP Builder* for generating HBPs, a series of examples were presented to illustrate the main usage and characteristics of *HBP Builder* package. In fact, it is very simple to get the *HBP Builder* running with the command line.

*python hbpbuilder-cg.py*

**Figure 3:**
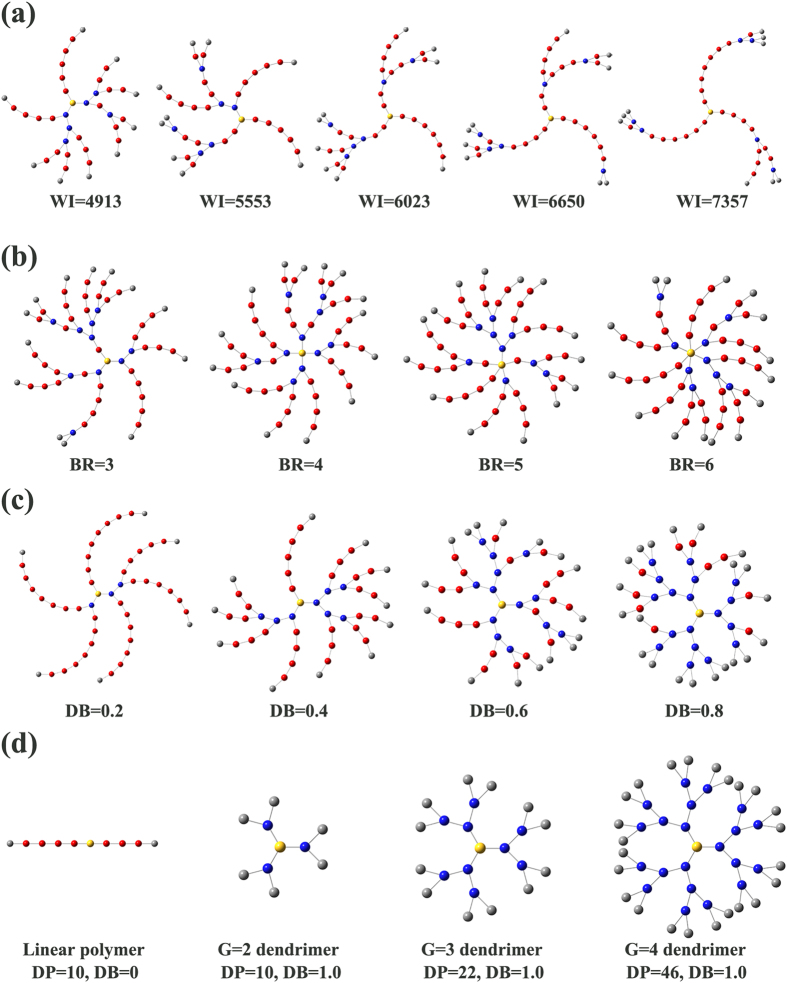
Different topological structures of HBPs generated by *HBP Builder_CG* module. Five topological structures of HBP with same *DP* (*DP* = 40), *DB* (*DB* = 0.4), *BR* (*BR* = 3) and different *WI* (**a**); Four topological structures with same *DP* (*DP* = 60), *DB* (*DB* = 0.4), and different *BR* (**b**); Four topological structures with same DP (DP = 50) and different DB (**c**); Linear polymer and dendrimers (**d**). Yellow bead represents root unit; blue bead represents dendritic unit; red bead represents linear unit; gray bead represents terminal unit.

Then it will pop up a dialogue as shown in Fig. 1b and all the parameters can be set through it. After all parameters setting was finished, user should press save button to save these input parameters and then press run button to run the program. Figure 3 present several examples generated by *HBP Builder_CG*, from which we can find *HBP Builder_CG* has several features:

1. HBPs is a mixture of many HBP molecules with a distribution of topological structure. As shown in Fig. 3a, *HBP Builder_CG* can quickly generate many HBPs with variable topological structures but with the same DP, DB, and BR. Based on this, we can investigate the effect of the topological structure on the property of individual HBP or the mixture of them.
2. In experiment, chemists can synthesize HBPs from the core molecules with multi functionalities, and the functionalities of the core molecules was defined as BR in our *HBP Builder*. By using *HBP Builder_CG*, we can generate HBPs with the same DP and DB, but with different BR (Fig. 3b). Based on this, we can investigate the effect of the core molecule structure on the property of individual HBP or the mixture of them.
3. It is well-known that the physichemical properties of HBPs, including the rheological properties, crystallization and melting behaviors, glass transition, thermal and hydrolytic degradation, optoelectronic properties, and so on, are highly dependent on DB. *HBP Builder_CG* can generate various structures with the same DP and BR, but with different DB (Fig. 3c). Based on these structures, we can furtherly disclose the relationship between *DB* and the properties of HBPs from a theoretical point of view.
4. In addition to the irregular HBPs, *HBP Builder_CG* also can quickly build well-defined structures, including linear polymers (BR = 2, DB = 0) with different compositions and molecular weight, and dendrimers (DB = 1.0) with different generations (Fig. 3d).

### The generation of Coarse-grained HBMCs

**Figure 4:**
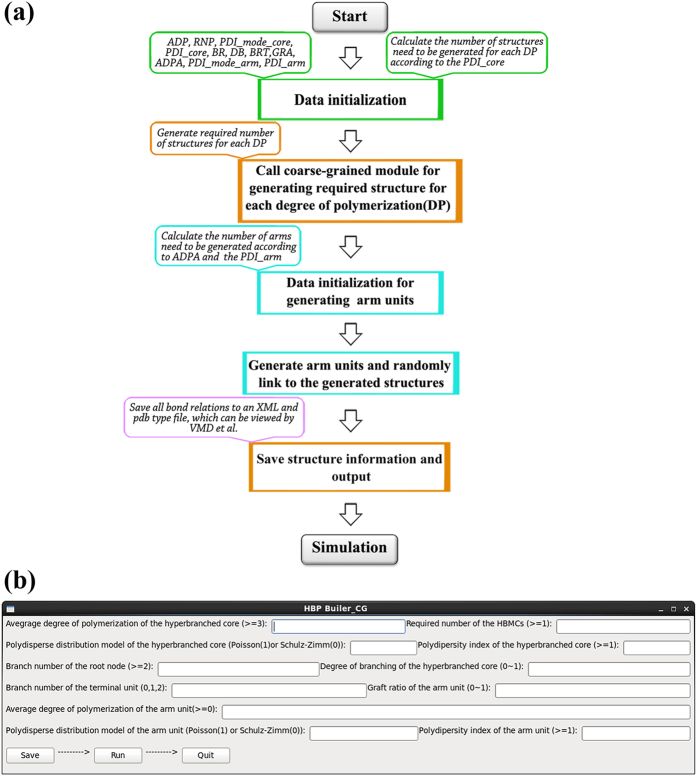
The flowchart of *HBP Builder_CG* for generating HBMCs. (**a**) The interactive input graphic interface of *HBP Builder_CG* for generating HBMCs (**b**).

HBMCs are composed of hyperbranched core and many linear arms, and are more complex than HBPs since the grafted linear arms also have polydispersity. Thus, the arm unit generation part was added to the *HBP Builder_CG* module to control the generation and the linking of arm units. In Fig. 4a, we present a flowchart to show the generation process of coarse-grained HBMCs through the *HBP Builder_CG* module. Likewise, an interactive graphic interface was also developed for building HBMCs (Fig. 4b). In this section, the characteristic parameters for describing linear arm units and the generation and linking process of arm units will be introduced explicitly.

### Input setting of the characteristic parameters

As shown in Fig. 4b, there are several characteristic parameters for arm unit need to be set except the parameters for hyperbranched core. The specific parameters added for HBMCs are as follows.

*Branch number of the terminal units* (BRT) is the number of the allowed linking position of each terminal unit.

*Graft ratio of the arm units* (GRA) is the ratio of the arm units bonded to terminal and linear units. If assign zero to *GRA*, HBMCs molecules without arms will be generated, which returns back to HBPs.

*Average degree of polymerization of linear arm* (ADPA) is the average DP of the arm units (DP_arm).

*Polydisperse distribution mode* (PDI_mode_arm) *and the polydispersity index* (PDI_arm) *of the linear arms* are the distribution mode and the width of the molecular weight of the arm units. Schulz-Zimm and Poisson distributions were also available for arm units.

### The generation and output of coarse-grained HBMCs

As shown in Fig. 4a, the generation process of coarse-grained HBMCs is based on the generation method of HBPs. That is, the generation process for hyperbranched core of HBMCs is identical to that of HBPs, which are not repeated here. And on this basis, after all required hyperbranched core generation has completed, the arm building subprogram will be called to generate the linear arm units with required number and length. Similar to the core generation, we firstly calculated the number of arms needs to be generated for each molecular weight according to the ADPA, PDI_mode_arm, PDI_arm and GRA of the arm units. Then all arm units will be generated one by one, and next randomly link it with the allowed joint units of the hyperbranched core one by one. After all arms are completely linked, we save molecular structure information for each HBMCs and packed all the molecules into one simulation box. Furthermore, the solvent molecules can be added in the same way as it used for HBPs. Finally, the xml and gro (and itp) type file were respectively outputted for DPD and coarse-grained molecular simulations (employed by Gromacs program package).

### The application example of coarse-grained HBMCs

**Figure 5:**
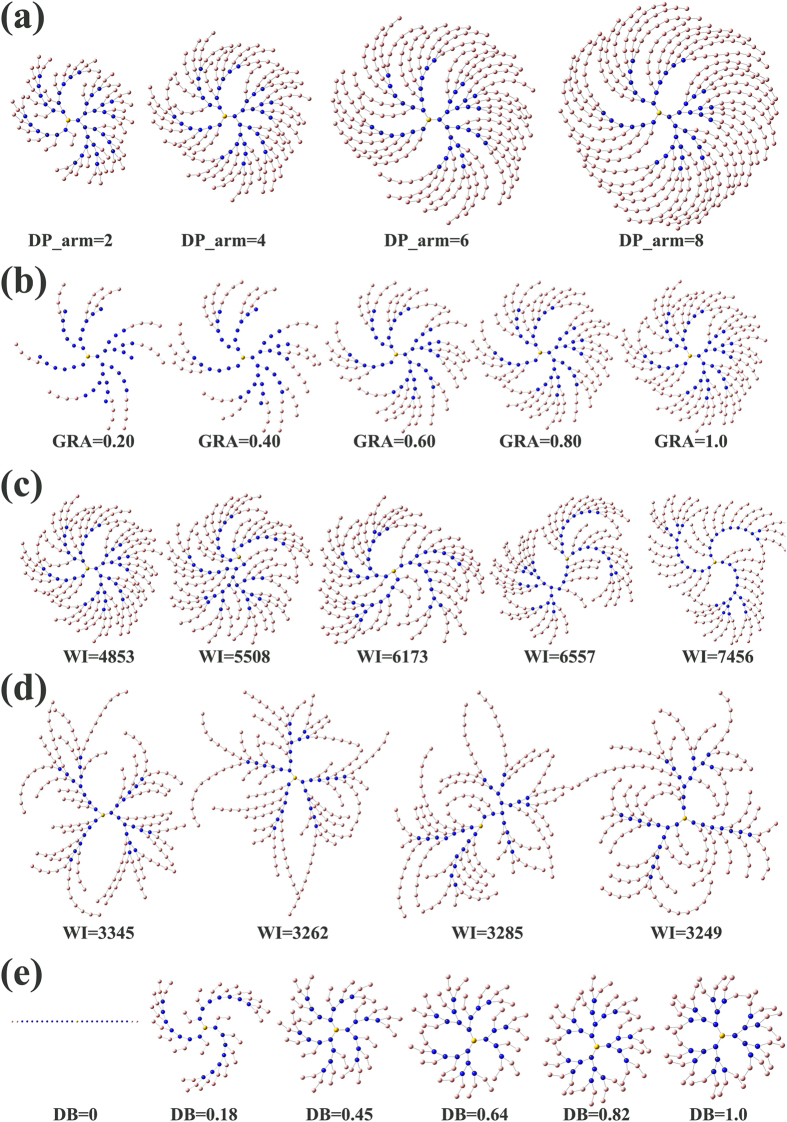
Different topological structures of HBMCs generated by *HBP Builder_CG module*. Four topological structures with same *DP* (*DP* = 40) of hyperbranched core and different arm length of linear arm (*DP_arm*) (**a**); Five topological structures with same hyperbranched core (*DP* = 40, *DB* = 0.40, *DP_arm* = 4) and different *GRA* (**b**); Five topological structures with same *DP* (*DP* = 40), *DB* (*DB* = 0.40), *DP_arm* (*DP_arm* = 4) and different WI of the hyperbranched core (**c**); Four topological structures with same *DP* (*DP* = 32), *DB* in the core (*DB* = 0.38), *ADPA* (*ADPA* = *5*) and different arm length distribution and WI in the core (**d**); Six topological structures with same *DP* (*DP* = 22), *DP_arm* (*DP_arm* = 2) and different *DB* in the core (*DB* = 0 is a ABA triblock copolymer, *DB* = 1.0 is a dendritic multi-arm copolymer with a dendrimer core (**e**). Yellow bead represents root unit; blue bead represents dendritic unit and linear unit of the hyperbranched core; pink bead represents linear arm units.

As shown in Fig. 5 and Figure S2 (supplementary information), we also present a series of examples to show the generated structures of HBMCs. It can be seen that the arm length, the graft ratio and PDI can be controlled flexibly. And the polydisperse HBMCs can be built conveniently through *HBP Builder_CG* module. Its main application range summarized below:

1. Amphiphilic HBMCs have been proved to be excellent self-assembly precursors. In experiment, HBMC with different arm length was designed to study the influence of the hydrophilic and hydrophobic ratio on the HBMC self-assembly behavior. In *HBP Builder_CG*, user can quickly build various HBMC structures with the same hyperbranched core but different length of arms (Fig. 5a).
2. The GRA is an important index to describe HBMC structure, and is also very important to regulate the properties of HBMCs. As shown in Fig. 5b, user can build various HBMC structures with the same hyperbranched core and different arms with variable GRAs through *HBP Builder_CG*. These structures can be used to disclose the GRA effect on the properties of HBMCs.
3. In the course of the HBMC self-assembly, the topological structure of the hyperbranched core has an effect on the self-assemblies. By using *HBP Builder_CG*, user can generate various HBMC structures (Fig. 5c) with the same arms (*GRA* and *DP_arm* are constant) and different topological hyperbranched cores (BR, DB, DP are the same, but the topology (WI) is different). Furthermore, the topology effect on the self-assembled structures can be investigated.
4. In the actual process of experiment, the arm length of the HBMCs always have a wide range of distribution. By using *HBP Builder_CG*, user can generate various HBMC structures (Fig. 5d) with the same DP and DB of the hyperbranched cores, and the average arm length (ADPA is constant), but different WI of the hyperbranched cores (DP are the same) and arm length with random distribution.
5. DB is often used in regulating the self-assembled morphologies of HBMCs. By using *HBP Builder_CG*, user can generate various HBMC structures (Fig. 5e) with the same arms (GRA and DP_arm are constant) and different *DB* of the hyperbranched cores (DP are the same). We can see HBMC turn to ABA triblock copolymer when DB decreases to 0. In the meanwhile, HBMC turn to dendritic multi-arm copolymer with a dendrimer core when DB increases to 1.0. Furthermore, the DB effect on the self-assembled structures can be studied.
6. In experiments, in solution or bulk state, it is a mixture of many HBMC molecules with a polydispersity of molecular weight in both hyperbranched core and arms, and a polydispersity of DB and a distribution of topological structure in the hyperbranched core. It is very complex. However, by using our *HBP Builder_CG*, the user can quickly generate a lot of polydisperse HBMCs just by inputting the average parameters of PDI (for the cores and arms) and DB (for the cores) through the keyboard (Figure S2). Thus, these simulation boxes can be used to disclose the PDI effect on the self-assembly behaviors of HBMCs. The molecular weight distributions of the generated HBPs/HBMCs by *HBP Builder_CG* agree well with the theoretical predications from Schulz-Zimm or Poisson distributions (Figure S3).

## The ***HBP Builder_AA*** function module

**Figure 6:**
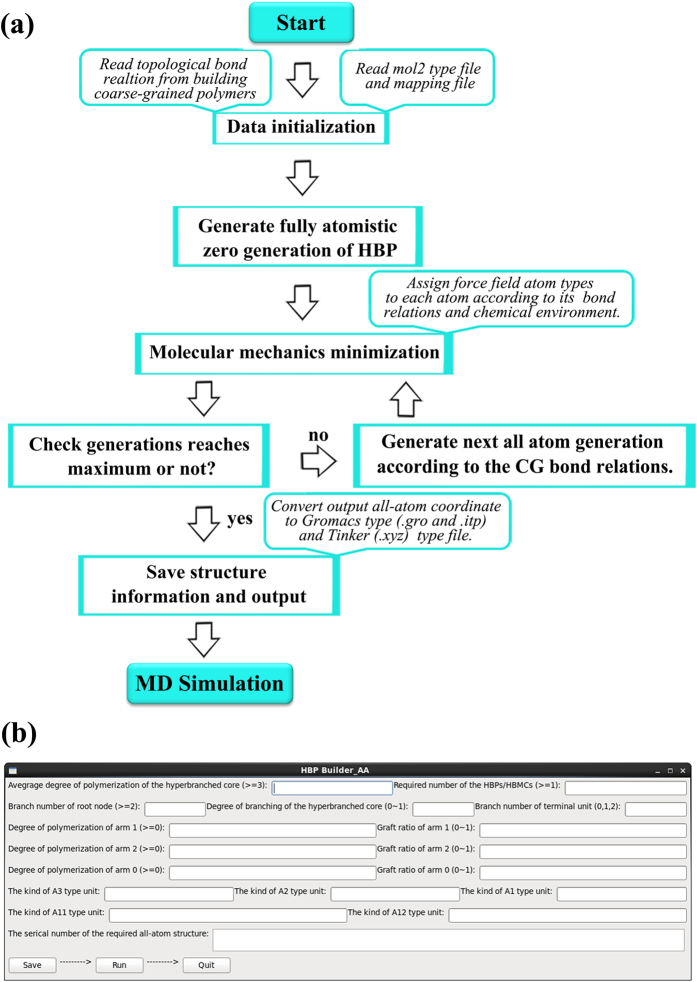
The flow chart of *HBP Builder_AA* module. (**a**) The interactive input graphic interface of *HBP Builder_AA* module (**b**).

The *HBP Builder_AA* is comprised of three subprograms: the input of the characteristic parameters, the coarse-grained HBPs/HBMCs reading or building, and the fully atomistic HBPs/HBMCs generation and output. We present the flowchart of the *HBP Builder_AA* module in Fig. 6a, which is explained in detail in this section.

### Input setting of the characteristic parameters

As shown in Fig. 6b, an interactive graphic interface was developed for *HBP Builder_AA* module. Thus all the related parameters can be set through it. The detailed notes on these parameters are explained as follows:

Except several parameters have been mentioned in *HBP Builder_CG* module, there are several characteristic parameters for describing the atomistic structure information. First of all, user should provide the name and types of all-atom repeat units. It worth noted that in some cases, several reactive place of the dendritic or the linear or the terminal units is not equal, which caused one unit has several possible connection ways, for instance, the two hydroxyl groups in the repeating unit of hyperbranched polyglycerol (HPG) are not equal, and there are two possible connection way. Thus, the possible connection ways of the repeating units with unequal reactivity should be defined in the dialogue box.

*A3_0*: the central core unit.

*A3_1*: the dendritic unit.

*A2_1*: the linear unit 1.

*A2_2*: the linear unit 2.

*A1*: the terminal unit.

*A11_1*: first type of the terminal unit with one arm unit.

*A11_2*: second type of the terminal unit with one arm unit.

*A12 type unit*: the terminal unit with two arms.

*L1 type unit*: the arm unit.

**Figure 7:**
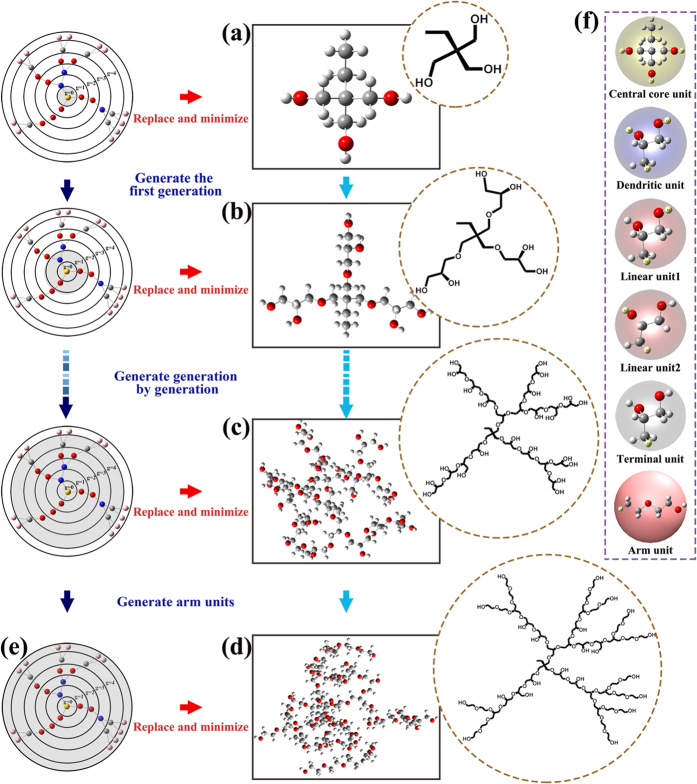
Scheme of the generation process of fully atomistic structure. First step for generating zero generation (**a**); Second step for generating first generation (**b**); The structure of HPG core (**c**); The structure of HPG-star-PEG (**d**) and its corresponding CG topology (**e**); The building units of HPG-star-PEG molecule (**f**).

Furthermore, the file name, the file location, and the index of the atom at joint points should be specified in a map file. Then, these possible connection ways will randomly appear in the generated polymers. The format of the map file is shown in Table S1 (supplementary information) and the corresponding structure segments can be seen in Fig. 7f.

*The serial number of the required all-atom structure* should be greater than zero and less than or equal to the required number of HBPs/HBMCs. Here a multi-line text input was created and the format is ‘N1 N2’. All structures for which index greater than or equal to N1 and less than or equal to N2 will be converted. If N1 equals N2 means only one structure required to be converted. User can add the serial number of other one or more structures in the next line.

Moreover, if HBMC with special topological structure is required, for example, considering the flexibility of the program, user can set the branch number of the terminal unit, and two arms with the same branch point can have their own component unit and graft ratio. The next several parameters need to be set:

*Degree of polymerization of arm 0*: the DP of the arm unit connected to linear units.

*Graft ratio of arm 0*: the graft ratio of the arm unit connected to linear units.

*Degree of polymerization of arm 1*: the DP of the arm unit connected to the first position of terminal unit.

*Graft ratio of arm 1*: the graft ratio of the arm unit connected to the first position of the terminal unit.

*Degree of polymerization of arm 2*: the DP of the arm unit connected to the second position of the terminal unit.

*Graft ratio of arm 2*: the graft ratio of the arm unit connected to the second position of the terminal unit.

### The back mapping process of *HBP Builder_AA*

In fact, there are two sources to obtain the topological bond relations in *HBP Builder_AA* module. One of them is read it from the generated coarse-grained HBP molecules. The other is build structures by their own special parameters as shown above. The coarse-grained HBPs/HBMCs generation process of this module is identical to the coarse-grained generation subprogram used above. When coarse-grained topological bond relations were obtained, the fine-graining subprogram will be called to convert them to fully atomistic structure. Here a back-mapping strategy was utilized to generate fully atomistic HBPs/HBMCs molecules according to the coarse-grained bond relations and the information of all-atom repeat units. Next, in order to obtain the reasonable molecular structure, the repeated replacement and energy minimization generation by generation process were realized in *HBP Builde_AA* module. Here, it should be noted that the main idea of this module is similar to that used in DBT^40^.

We take HPG-star-PEG as an example to illustrate the mapping process. The explicit process is as follows:

1. A coarse-grained HPG-star-PEG was generated and each CG unit was assigned to its corresponding generation number, as shown in Fig. 7e. Next the zero-generation of the HBMC molecules was replaced with the preset all-atom units (Fig. 7f). In *HBP Builder*, the preset all-atom units were written in mol2 type. Meanwhile, the hydrogen atoms which to be deleted in the linker positions were listed in the map file (the yellow atom in Fig. 7f and Table S1).
2. Assigning atom type of force field to each atom of the generated molecule according to the bond relations and chemical environment of each atom. Currently only a part of atom types of OPLS_AA force field has been incorporated in *HBP Builder_AA* module. Nonetheless, it does not matter, because other atom types can be easily added to *HBP Builder_AA* module. Then energy minimization of molecular mechanics was employed to the generated molecule until satisfy the convergence criterion. Next, we saved structure information of the optimized molecule for the next generation stage.
3. We added first fully atomistic generation to the above optimized zero-generation structure according to the coarse-grained bond relations (Fig. 7a). Meanwhile, we linked discrete all-atom units and deleted the redundant hydrogen atoms. After that, succeeding steps will be performed in order, store the atom coordinates and bond relations, assign atom types, optimize the generated structure and save the structure information (Fig. 7b). As mentioned earlier, the branchpoint of dendritic unit, linear unit and terminal unit are not equivalent in some cases, user should input all the possibility and provide the label of the hydrogen atom of the connection points. As shown in Fig. 7f, the HPG core has two possible linear units. So the coarse-grained linear units will be substituted for these two all-atom units randomly.
4. Repeat the third step until the entire HPG core was completely replaced and optimized (Fig. 7c).
5. Then, the coarse-grained arm unit will be replaced with the PEG unit. The process is similar to that generation process of HPG core, we link discrete all-atom units and delete the redundant hydrogen atoms, assign atom types, optimize the generated structure and save the structure information (Fig. 7d).
6. Finally, two types of files were outputted for MD simulations after the structure generation. They are the xyz type file for Tinker program and gro (and itp) type file for Gromacs package. User can add additional output file types to meet their own needs easily by minor revisions of the program. In order to make the output more intuitively, the graphic library OpenGL was linked in *HBP Builder* program. Thus the final structure can be directly observed through the display window. Of course, user can also view it through other visualization software such as VMD etc.^38^.

### The application example of *HBP Builder_AA*

The atomistic insight is very important when special interactions need to be considered, for example, the noncovalent interactions like hydrogen bonds or specific chemical interactions between polymers, drugs, DNAs or proteins. Here, to show the fully atomistic structures generated through *HBP Builder_AA* module, a series of examples were presented.

**Figure 8 f8:**
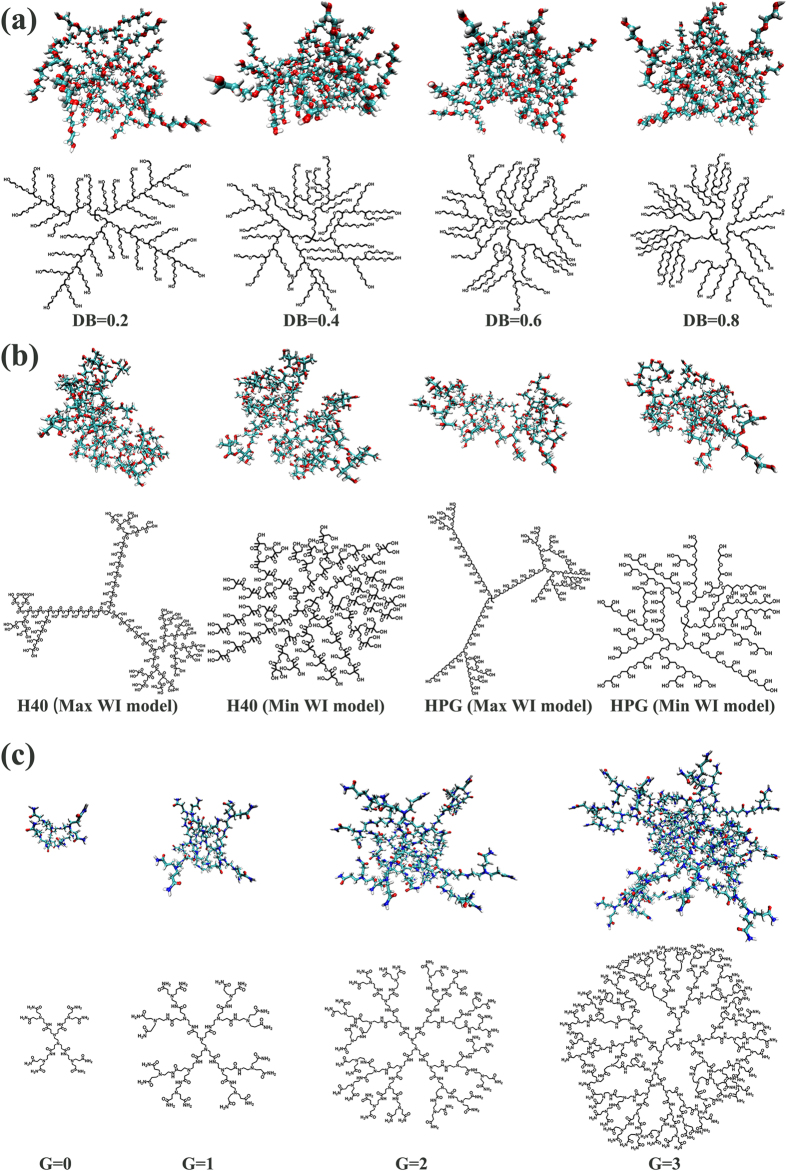
Different fully atomistc structures of HBPs generated by *HBP Builder_AA module*. HPG-star-PEG with different DB (**a**); Max *WI* and Min *WI* model molecules for H40 and HPG (**b**); PAMAM dendrimers with different generation (**c**). In each figure, the upper one is the CPK model, and the lower one is the corresponding structural formula.

1. User can build a series of fully atomistic HBMC structures with different *DB* values in the hyperbranched core. As shown in Fig. 8a, several HPG-star-PEGs with different DB values were generated through *HBP Builder_AA*. Furthermore, these model molecules can be used to study the dependency of unimolecular properties on the DBs.
2. User can build different fully atomistic HBPs based the same coarse-grained structures. As shown in Fig. 8b, two fully atomistic structures for H40 and HPG molecules were generated through *HBP Builder_AA*. Among them, Max WI models of H40 and HPG (or Min WI model of H40 and HPG) have the same coarse-grained structure, however, the fully atomistic structures are totally different. One is the hyperbranched polyester (H40), and the other is hyperbranched polyether (HPG). On the basis of these structures, user can investigate the properties of different type of all-atom HBP molecules with same coarse-grained topology.
3. User also can build fully atomistic structures for regular dendrimers. The *HBP Builder_CG* can generate coarse-grained dendrimers. Then after inputting the repeat unit information, as shown in Fig. 8c, the fully atomistic structures of PAMAM dendrimers from zeroth to third generation were built through *HBP Builder_AA*. Thus, the properties of these dendrimers can be studied through molecular simulation.

## The performance of ***HBP Builder***

To illustrate the high efficiency of *HBP Builder*, Table S2 (supplementary information) summarizes several systems and the corresponding computational time required to generate them. All these examples[Fig f8]were run on an Intel(R) Core(TM) Quad i7-3610QM CPU with 2.30 GHz clock rate and 8 Gb memory, and implemented under the openSUSE 13.2 embedded operating system. Table S2 shows that with the increment of *DP* and *RNP*, the computational time is increased gradually. Overall, however, *HBP Builder* has a high efficiency to generate the user required structures.

## Conclusions and Outlook

In this work, *HBP Builder* program for building HBPs/HBMCs with different degree of polymerization, degree of branching and polydispersity index is presented. Several parameters for characterizing the features of hyperbranched molecules are introduced to control over the final topological structures. An unordered tree-inclusion matching model is used in the program to eliminate the repetitive structures. Furthermore, fully atomistic structures are obtained through a back-mapping scheme, which generated and optimized the HBPs/HBMCs structures generation by generation. We have generated a series of coarse-grained and fully atomistic structures to illustrate the application and validation of *HBP Builder*. These results have been shown that our tool is very useful and can be further extended to build HBPs/HBMCs of any desired degree of polymerization, degree of branching and polydispersity index by providing structure parameters and user defined building units. In the meanwhile, *HBP Builder* can also be used to build linear block copolymers, dendrimers or dendritic multi-arm copolymers when set degree of branching in the cores to 0 or 1.0. The modular architecture making *HBP Builder* easier to add new molecule types and force fields and also reuse it as a part of other programs. We have reasons to believe *HBP Builder* will be a promising tool to construct model molecules for studying of HBPs/HBMCs interacts with the polymers, drug molecules, protein and nucleic acid, and HBMCs self-assembly in solution.

## Additional Information

**How to cite this article**: Yu, C. *et al.* *HBP Builder*: A Tool to Generate Hyperbranched Polymers and Hyperbranched Multi-Arm Copolymers for Coarse-grained and Fully Atomistic Molecular Simulations. *Sci. Rep.* **6**, 26264; doi: 10.1038/srep26264 (2016).

## Supplementary Material

Supplementary Information
