# Supplementary material for: HBP Builder: A Tool to Generate Hyperbranched Polymers and Hyperbranched Multi-Arm Copolymers for Coarse-grained and Fully Atomistic Molecular Simulations
Source: Sci Rep. 2016 May 18;6:26264. doi: 10.1038/srep26264 (PMC4870682; doi:10.1038/srep26264)
Supplement: Supplementary Information [file srep26264-s1.pdf]

HBP Builder: A Tool to Generate Hyperbranched Polymers and Hyperbranched Multi-Arm Copolymers for Coarse-grained and Fully Atomistic Molecular Simulations

Chunyang Yu, Li Ma, Shanlong Li, Haina Tan, Yongfeng Zhou* and Deyue Yan

School of Chemistry and Chemical Engineering, State Key Laboratory of Metal Matrix Composites, Shanghai Jiao Tong University, 800 Dongchuan Road, Shanghai 200240, P. R. China

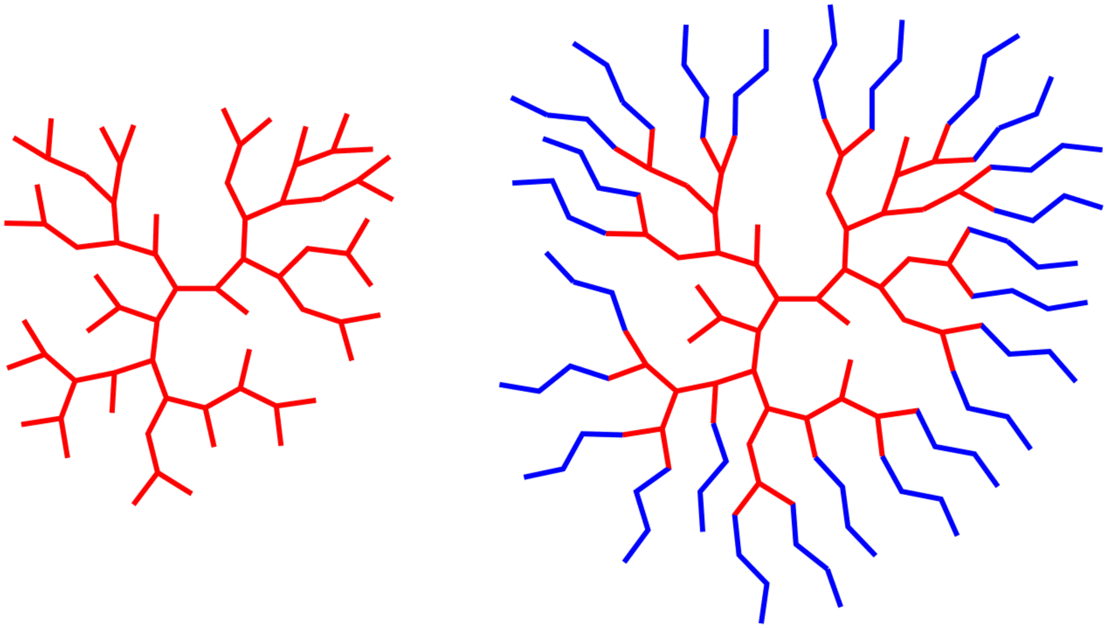

Figure S1. Schematic representation of HBPs and HBMCs. Red blocks are hydrophobic hyperbranched core while blue ones are hydrophilic linear arms.

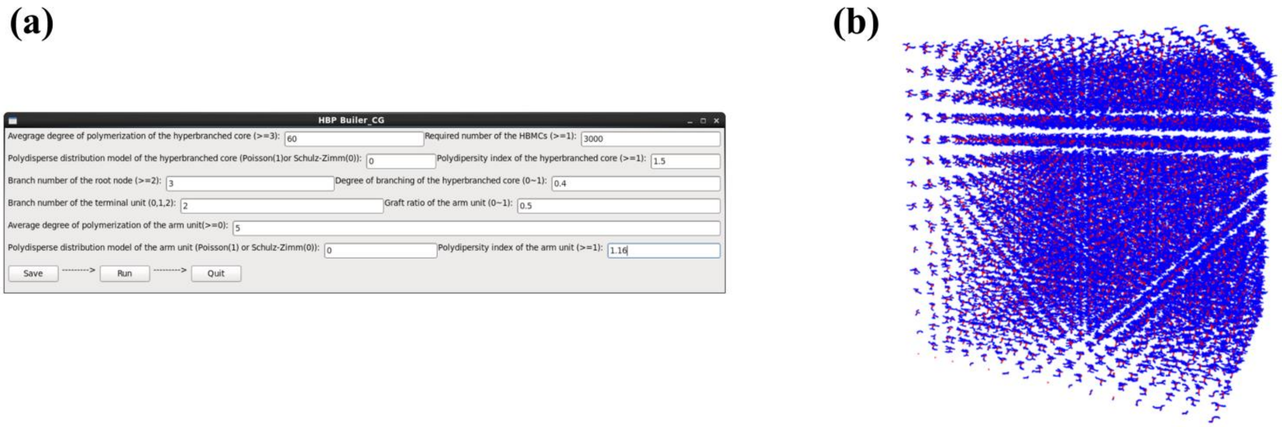

Figure S2. Simulation box with various HBMCs structures generated by HBP Builder_CG. The input dialogue (a); The generated simulation box with polydisperse HBMCs (b).

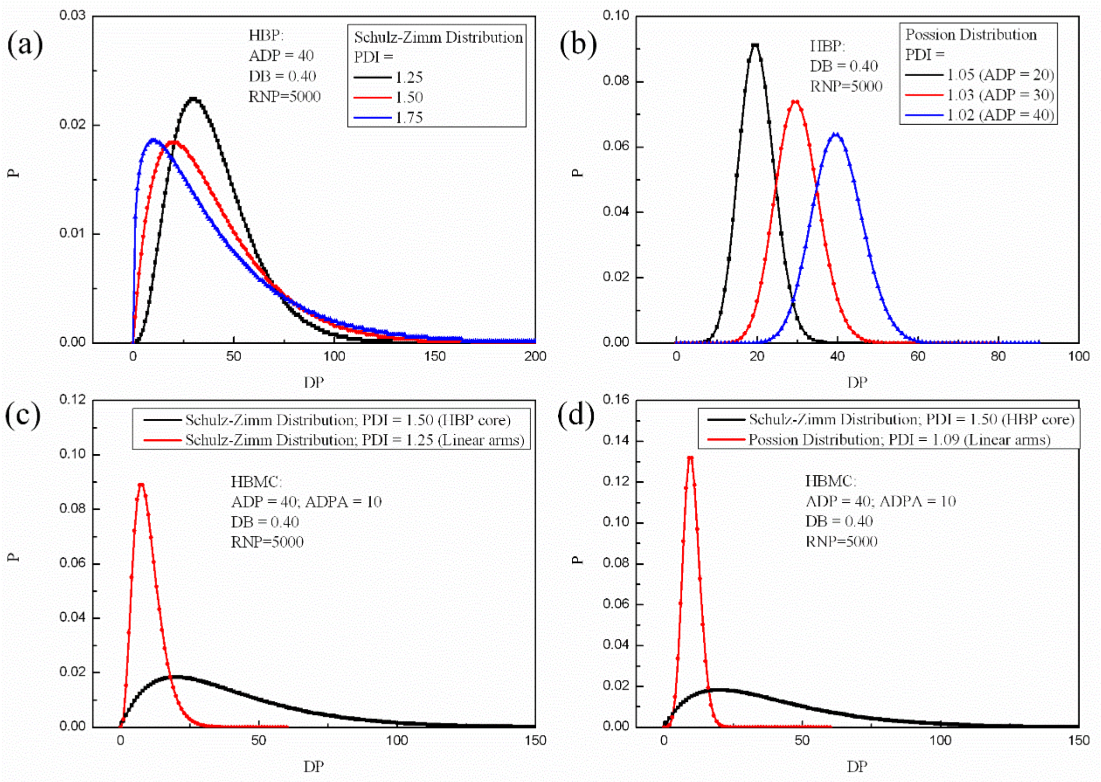

Figure S3. Schulz-Zimm distributions of HBPs (a); Possion distributions of HBPs (b); Schulz-Zimm distributions of HBMCs (c); Schulz-Zimm/Possion distributions of HBMCs. In each figure, the solid lines represent the calibration curves and the points represent the distribution generated by HBP Builder. ADP represents the average degree of polymerization of HBP; ADPA represents the average degree of polymerization of linear arms; RNP represents the required number of the polymers; DP represents the degree of polymerization; P represents the normalized distribution probability. As shown in Figures S3a and S3b, all the HBPs generated by HBP Builder (data points) totally conform to the theoretical predications from Schulz-Zimm or Poisson distribution (continuous lines). Meanwhile, both the arms and the HBP cores of HBMCs generated by HBP builder also conform to the theoretical predications from Schulz-Zimm or Poisson distribution very well (Figures S3c and S3d).

Table S1. The map file of HPG-star-PEG.

Type	File location	Joint point number	Atom index
A3_0	mol2//a30.mol2	3	19 21 23
A3_1	mol2//a3.mol2	3	13 10 12
A2_1	mol2//a2.mol2	2	13 10
A2_2	mol2//a2.mol2	2	13 12
A1_1	mol2//a2.mol2	1	13
A11_1	mol2//a2.mol2	2	13 10
A11_2	mol2//a2.mol2	2	13 12
A12	mol2//a2.mol2	3	13 10 12
L1	mol2//peg.mol2	1	3

Table S2. The Running time(t) of several studied systems.

RNP	DP	PDI_core PDI_arm	BRT	DB	GRA	DP_arm	t (s)
10	22	1.0/1.0	3	0.4	1.0	5	<1
10	46	1.0/1.0	3	0.5	1.0	5	<1
100	66	1.0/1.0	3	0.6	1.0	5	1
1000	40	1.0/1.0	3	0.4	1.0	10	27
1000	40	1.5/1.2	3	0.4	1.0	10	26
1000	40	1.5/1.2	4	0.4	1.0	10	27
1000	60	1.5/1.2	3	0.4	1.0	10	52
1000	60	1.5/1.2	4	0.4	1.0	10	50
1000	80	1.5/1.2	3	0.4	1.0	10	88
1000	80	1.5/1.2	4	0.4	1.0	10	84
2000	80	1.5/1.2	3	0.4	1.0	20	421
3000	80	1.5/1.2	3	0.4	1.0	20	900
